# Comparison of efficacies of haploidentical transplantation and matched sibling donor transplantation in treating T‐cell lymphoblastic lymphoma

**DOI:** 10.1002/cam4.5786

**Published:** 2023-03-29

**Authors:** Ruowen Wei, Jun Fang, Wei Shi, Xuan Lu, Yingying Wu, Shan Jiang, Ao Zhang, Shanshan Liao, Chunxia Qin, Guohui Cui, Linghui Xia

**Affiliations:** ^1^ Institute of Hematology, Union Hospital, Tongji Medical College Huazhong University of Science and Technology Wuhan China; ^2^ Department of Nuclear Medicine, Union Hospital, Tongji Medical College Huazhong University of Science and Technology Wuhan China

**Keywords:** allogeneic hematopoietic stem cell transplantation, haploidentical, matched sibling donor, PET/CT, T‐cell lymphoblastic lymphoma

## Abstract

**Objective:**

To investigate the differences in efficacy and safety between haploidentical donor hematopoietic stem cell transplantation (HID‐HSCT) and matched sibling donor HSCT (MSD‐HSCT) in patients with T‐cell lymphoblastic lymphoma (T‐LBL).

**Methods:**

In this retrospective analysis, we enrolled 38 patients who had undergone allogeneic HSCT at our institution between 2013 and 2021. The study participants included 28 patients who underwent HID‐HSCT and 10 patients who underwent MSD‐HSCT. We compared the patient characteristics and treatment effectiveness and safety between the two groups and evaluated potential prognostic variables for patients with T‐LBL.

**Results:**

The median follow‐up durations in the HID‐HSCT and MSD‐HSCT groups were 23.5 (range: 4–111) and 28.5 (range: 13–56) months, respectively. All patients showed full‐donor chimerism after hematopoietic stem cell transplantation (HSCT). Except for two patients in the HID‐HSCT cohort who developed poor graft function, all patients showed neutrophil and platelet engraftments after HSCT. The cumulative incidences of grades III–IV acute graft‐versus‐host disease were 37.5% and 28.57% in the HID‐HSCT and MSD‐HSCT groups, respectively (*p* = 0.84). The cumulative incidences of limited (34.13% vs. 28.57%, *p* = 0.82) and extensive (31.22% vs. 37.50%, *p* = 0.53) chronic graft‐versus‐host disease did not differ between the two cohorts. In the HID‐HSCT and MSD‐HSCT cohorts, the estimated 2‐year overall survival rates were 70.3% (95% confidence interval [CI]: 54.9%–90.0%) and 56.2% (95% CI: 31.6%–100%), respectively (*p* = 1.00), and the estimated 2‐year progression‐free survival (PFS) rates were 48.5% (95% CI: 32.8%–71.6%) and 48.0% (95% CI: 24.6%–93.8%), respectively (*p* = 0.94). Furthermore, the Cox proportional‐hazards model showed that a positive positron emission tomography/computed tomography (PET/CT) status before HSCT in patients who had completed chemotherapy was an independent risk factor for PFS in the multivariate analysis (*p* = 0.0367).

**Conclusion:**

This study showed that HID‐HSCT had comparable effectiveness and safety to MSD‐HSCT in treating T‐LBL. HID‐HSCT could serve as an alternate treatment option for T‐LBL in patients without an eligible identical donor. Achievement of the PET/CT‐negative status before HSCT may contribute to better survival.

## 1 INTRODUCTION

T‐cell lymphoblastic lymphoma (T‐LBL) is an extremely aggressive type of non‐Hodgkin's lymphoma. Its incidence rate is 0.1 per 100,000 adolescents and young adults and 0.4 per 100,000 children <15 years.^1^ T‐LBL and T‐cell acute lymphoblastic leukemia (T‐ALL) have similar morphologic and immunologic features and have been called “T lymphoblastic lymphoma/leukemia (T‐LBL/ALL)” by the World Health Organization (WHO) since 2008.^2^ The key distinction between them is the degree of bone marrow involvement: T‐LBL, <25% (or 20% according to WHO); and T‐ALL, ≥25%.^3^ The prognosis of adults with T‐LBL is poor, with a low complete remission (CR) rate and overall survival (OS) ranging from 11 to 17 months.^4^, ^5^, ^6^, ^7^ Currently, T‐LBL has no standard treatment. Although high CR rates may be achieved with ALL‐like therapy and the modified Berlin–Frankfurt–Münster‐90 (BFM‐90) regimen, approximately 30% of patients develop relapse.^8^, ^9^, ^10^

Hematopoietic stem cell transplantation (HSCT) may improve the prognosis of patients with T‐LBL; however, whether allogeneic HSCT (allo‐HSCT) or autologous HSCT (auto‐HSCT) is more favorable remains debatable. Allo‐HSCT may yield similar survival outcomes as auto‐HSCT.^11^, ^12^ Allo‐HSCT is associated with a relatively lower relapse rate but a higher transplantation‐relative mortality (TRM). While the safety of auto‐HSCT is better, the relapse rate is higher. A conclusion regarding which one is better for T‐LBL has not been established. Allo‐HSCT has been shown to reduce the risk of relapse in T‐cell non‐Hodgkin lymphoma.^13^, ^14^ However, only 30% of patients have human leukocyte antigen (HLA)‐identical sibling donors,^15^ most patients need alternative donors. Currently, haploidentical donor HSCT (HID‐HSCT) is widely used to treat hematological malignancies and has been undergoing rapid advancements in China.^16^ HID‐HSCT and matched sibling donor (MSD‐HSCT) have comparable therapeutic effects in treating ALL.^17^, ^18^, ^19^ However, although HID‐HSCT treated T‐LBL with good outcomes, no studies on the comparison between HID‐HSCT and MSD‐HSCT have been performed.^20^, ^21^ Their advantages and disadvantages are yet unknown. Therefore, the aim of this study was to compare the effectiveness and safety of MSD‐HSCT and HID‐HSCT and investigate prognostic factors for patients with T‐LBL who undergo HSCT.

## 2 MATERIALS AND METHODS

### 2.1 Study population

This was a single‐center, retrospective analysis of 38 patients with T‐LBL who had undergone allo‐HSCT between May 2013 and September 2021. T‐LBL was diagnosed according to the 2016 criteria by WHO.^22^ The examinations mainly included bone marrow biopsy and morphologic assessment, flow cytometry of bone marrow aspirates, immunohistochemistry, and positron emission tomography/computed tomography (PET/CT). All patients underwent HSCT after showing CR, partial remission, or stable disease with the BFM‐90 or hyper‐CVAD chemotherapy regimen. The study protocol was approved by the ethics committee of the Union Hospital, Tongji Medical College, Huazhong University of Science and Technology. All patients provided informed consent. The study was performed in accordance with the tenets of the Declaration of Helsinki.

### 2.2 Donor–recipient human leukocyte antigen typing

All donor–recipient pairings were matched using HLA serologic typing for class I antigens and HLA molecular typing for the *DRB1* and *DQB1* loci. A high‐resolution molecular approach was used to verify the validities of HLA‐A, HLA‐B, HLA‐C, HLA‐*DRB1*, and HLA‐DQB1.

### 2.3 Conditioning regimens

Conditioning regimens were provided for radiotherapy and chemotherapy. For patients who underwent HID‐HSCT, conditioning regimens were as follows^1^: total body irradiation (TBI, 10 Gy) with partial shielding of the lungs and eyes and idarubicin (IDA, 12 mg/m^2^/day on Days −6 to −5) or etoposide (VP‐16, 125 mg/m^2^ i.v. twice daily on Day −5), followed by cyclophosphamide (CY, 60 mg/kg/day on Days −3 to −2); total marrow irradiation (TMI, 8 Gy) and IDA (12 mg/m^2^/day i.v. on Days −6 to −5) or VP‐16 (125 mg/m^2^ i.v. twice daily on Day −5), followed by CY (60 mg/kg/day on Days −3 to −2); and^2^ VP‐16 plus the BUCY regimen, containing busulfan (3.2 mg/kg/day i.v. on Days −9 to −7) and CY (60 mg/kg/day on Days −3 to −2). For patients who underwent MSD‐HSCT, conditioning regimens were as follows^1^: TMI or TBI plus IDA or VP‐16 plus CY (same dosages as aforementioned)^2^; VP‐16 plus the BUCY regimen (same dosages as aforementioned); and^3^ TBF regimen, containing thiotepa (5 mg/kg/day on Days −7 to −6) plus Bu (3.2 mg/kg/day i.v. on Days −5 to −3) and fludarabine (40 mg/m^2^/day on Days −5 to −2). Hospitalized patients were housed in a single room with highly efficient air‐filtering devices. Supportive care was provided as previously described.^23^

### 2.4 Stem cell harvest

All donors were subcutaneously administered with 8–10 μg/kg of recombinant human granulocyte colony‐stimulating factor (rhG‐CSF) daily to mobilize stem cells from 3 days before HSCT to the last day of HSCT. Basing on the target count for CD34^+^ cells set at above 3 × 10^6^/kg of the recipient weight, rhG‐CSF was applied for an average of 5 days. rhG‐CSF‐primed peripheral blood stem cells and/or bone marrow stem cells were collected on the fourth to fifth day after rhG‐CSF administration and transfused to the recipients on the same day without manipulation. A one‐day extension of mobilization and transfusion period would be required if the count of CD34^+^ cells did not reach the target level.

### 2.5 Graft‐versus‐host disease prophylaxis

For patients who underwent HID‐HSCT, graft‐versus‐host disease (GVHD) prophylaxis comprised tacrolimus (FK‐506) or cyclosporine A (CsA), methotrexate (MTX), mycophenolate (MMF), basiliximab, and anti‐lymphocyte globulin (ALG) or anti‐thymocyte globulin (ATG). FK‐506 (0.05 mg/kg, twice daily) was administered orally from Day −1. CsA (5 mg/kg, twice daily) was administered intravenously from Day −1 until oral medications were tolerated by patients. Adjustments to the dosage of CsA or FK506 were based on plasma target levels of 150–250 or 7–15 ng/mL, respectively. The dosage of CsA or FK506 was tapered by 20% monthly and discontinued around 6 months after HSCT in cases without GVHD. MTX was administrated intravenously at a dosage of 20 mg/m^2^/day on Day +1, followed by a dosage of 15 mg/m^2^/day on Days +3, +6, and +11. MMF (7.5 mg/kg, twice daily) was administered orally on Day −9, followed by the half dosage until Day +30, and terminated on Day +60 if GVHD was absent. Basiliximab (20 mg/kg/day, i.v.) was administrated on Days 0 and +4. ALG (25–30 mg/kg/day) and ATG (3 mg/kg/day) were administrated intravenously on Days −3 to −1. For patients who underwent MSD‐HSCT, GVHD prophylaxis comprised CsA and MTX with or without MMF (same dosages as aforementioned).

### 2.6 Regimen‐related toxicities

Regimen‐related toxicities were assessed according to the Bearman toxicity criteria.^24^ Organ damage resulting from GVHD and/or infection was excluded from the evaluation.

### 2.7 Therapeutic evaluation and study definition

Eligible patients had undergone at least three PET/CT examinations: at diagnosis and during and after chemotherapy (before HSCT). Response was assessed as CR, partial remission, stable disease, or progressive disease according to the 2014 Lugano Classification.^25^ The Deauville scale was used to determine the PET‐negative (Deauville ≤3)/PET‐positive (Deauville >3) status. Hematopoietic reconstitution and chimerism were evaluated as previously described.^26^ ABO mismatch was classified into three types, depending on the transfusion strategy.^27^ Primary poor graft function (PGF) was defined as Sun et al. described earlier.^28^ The diagnosis and the grade of acute GVHD (aGVHD) and chronic GVHD (cGVHD) were assessed according to the Glucksberg–Seattle criteria and the National Institutes of Health consensus.^29^, ^30^, ^31^, ^32^ Progression‐free survival (PFS) was defined as the duration between diagnosis and relapse, death from any cause, or the final follow‐up day. OS was defined as the duration between diagnosis and death from any cause or the last follow‐up day. Death without relapse or progressive disease after allo‐HSCT was defined as TRM. OS, PFS, and cumulative incidence of relapse (CIR) were the primary endpoints of the study. TRM and cumulative incidence of GVHD were the secondary endpoints.

### 2.8 Statistical analysis

Descriptive statistics were performed for demographic and clinical patient characteristics. Continuous variables were compared using the Mann–Whitney *U* test. Classification variables were assessed using the chi‐square or Fisher's exact test. The Kaplan–Meier estimator with the log‐rank test was used to estimate PFS and OS. Potential factors affecting OS and PFS were explored using univariate and multivariate analyses of the Cox proportional‐hazards model. Cumulative incidence curves with Fine & Gray model were used to estimate the probabilities of TRM, GVHD, and relapse. TRM was considered as a competing risk of relapse. Relapse and death without GVHD were competing events for aGVHD and cGVHD. All *p*‐values were two‐sided. A *p*‐value <0.05 was considered to indicate statistical significance. All statistical analyses were performed using SPSS version 25.0 (SPSS Inc.) and R Studio (version 1.4.1106).

## 3 RESULTS

### 3.1 Patient characteristics

Table 1 shows detailed patient information. The median follow‐up durations in the HID‐HSCT and MSD‐HSCT groups were 23.5 (range: 4–111) and 28.5 (range: 13–56) months, respectively. Table 2 shows the clinical outcomes of HSCT.

**TABLE 1:** Basic information of allo‐HSCT patients.

Characteristics	HID‐HSCT	MSD‐HSCT	*p*‐value
Number of patients	28	10	—
Median age, year (range)	23.5 (14–43)	28.5 (19–46)	0.023
Gender			1.000
Male	21 (75.0)	7 (70.0)	
Female	7 (25.0)	3 (30.0)	
B symptom			0.690
No	19 (67.9)	8 (80.0)	
Yes	9 (32.1)	2 (20.0)	
LDH at diagnose			0.468
Normal	12 (42.9)	6 (60.0)	
Elevated	16 (57.1)	4 (40.0)	
Ann Arbor			0.263
III	0 (0)	1 (10.0)	
IV	28 (100)	9 (90.0)	
Mediastinal mass at diagnosis			1.000
No	9 (32.1)	3 (30.0)	
Yes	19 (67.9)	7 (70.0)	
ECOG			0.714
0	15 (53.6)	4 (40.0)	
1–2	13 (46.4)	6 (60.0)	
IPI			0.714
1–2	13 (46.4)	6 (60.0)	
3–4	15 (53.6)	4 (40.0)	
HCT‐CI			0.666
0	23 (82.1)	9 (9.0)	
1–2	5 (17.9)	1 (10.0)	
MRD before HSCT			1.000
Negative	27 (96.4)	10 (100)	
Positive	1 (3.6)	0 (0)	
PET/CT evaluation before HSCT			0.800
CR	23 (82.2)	9 (90.0)	
PR	2 (7.1)	1 (10.0)	
SD	3 (10.7)	0 (0)	
PET/CT negative/positive before HSCT			1.000
Negative	15 (53.6)	6 (60.0)	
Positive	13 (46.4)	4 (40.0)	
Donor/recipient gender			0.890
Male to male	13 (46.4)	5 (50.0)	
Male to female	6 (21.4)	2 (20.0)	
Female to female	1 (3.6)	1 (10.0)	
Female to male	8 (28.6)	2 (20.0)	
ABO match			0.204
Matched	14 (50.0)	5 (50.0)	
Major mismatched	6 (21.4)	4 (40.0)	
Minor mismatched	7 (25.0)	0 (0)	
Bi‐directional	1 (3.6)	1 (10.0)	
Donor/recipient CMV serologic status			—
Negative to negative	28 (100)	10 (100)	
Donor/recipient EBV serologic status			—
Negative to negative	28 (100)	10 (100)	
Stem cell source			0.168
PBSC	22 (78.6)	10 (100)	
PBSC + BMSC	6 (21.4)	0 (0)	
Conditioning regimens			—
TBI + IDA or VP‐16 + CY	3	1	
TMI + IDA or VP‐16 + CY	16	7	
VP‐16 + BUCY	9	1	
TBF	0	1	
GVHD prophylaxis			—
CsA + MTX + MMF + basiliximab + ATG	4	0	
FK‐506 + MTX + MMF + basiliximab + ATG or ALG	24	0	
CsA + MTX	0	7	
CsA + MTX + MMF	0	2	
FK506 + MTX	0	1	
Median time from diagnosis to HSCT, months (range)	6 (3–13)	6 (4–8)	0.401
Median nucleated cells, ×10^8^/kg (range)	13.77 (8.62–32.08)	11.645 (8.42–17.60)	0.034
Median CD34^+^ cells, ×10^6^/kg (range)	6.820 (3.567–14.160)	5.845 (3.040–10.370)	0.257
Median follow‐up time, months (range)	23.5 (4–111)	28.5 (13–56)	—

*Note*: Data are presented as *n* (%) unless indicated otherwise.

Abbreviations: ALG, anti‐lymphocyte globulin; ATG, anti‐thymocyte globulin; BM, bone marrow stem cell; BUCY, busulfan (BU) plus CY; CR, complete remission; CsA, cyclosporine A; CY, cyclophosphamide; ECOG, Eastern Cooperative Oncology Group score; FK‐506, tacrolimus; HCT‐CI, hematopoietic cell transplantation‐specific comorbidity index; HID, haploidentical donor; HSCT, hematopoietic stem cell transplantation; IDA, idarubicin; IPI, the International Prognostic Index; MMF, mycophenolate; MRD, minimal residual disease assessed by flow cytometry; MSD, matched related donor; MTX, methotrexate; PBSC, peripheral blood stem cell; PET/CT negative, Deauville score ≤3; PR, partial response; SD, stable disease; TBF, thiotepa plus Bu and fludarabine; TBI, total body irradiation; TMI, total marrow irradiation; VP‐16, etoposide.

**TABLE 2:** Clinical outcomes of allo‐HSCT patients.

Outcome	HID‐HSCT	MSD‐HSCT	*p*‐value
Neutrophil engraftment rate	28 (100)	10 (100.0)	—
Median time of neutrophil engraftment, days (range)	11 (9–24)	11 (10–15)	0.832
Platelet engraftment rate	26 (92.9)	10 (100.0)	1.000
Median time of platelet engraftment, days (range)	11 (7–24)	10 (9–14)	0.166
CMV reactivation	9 (32.1)	2 (20.0)	0.690
EBV reactivation	5 (17.9)	2 (20.0)	1.000
Pneumonia	10 (35.7)	2 (20.0)	0.453
Oropharyngeal mucositis	12 (42.9)	4 (40.0)	1.000
Hemorrhagic cystitis	3 (10.7)	0 (0)	0.552
aGVHD grade			—
No	10 (35.7)	5 (50.0)	
I–II	12 (42.9)	3 (30.0)	
III–IV	6 (21.4)	2 (20.0)	
cGVHD grade			—
No	14 (50.0)	5 (50.0)	
Limited	7 (25.0)	2 (20.0)	
Extensive	7 (25.0)	3 (30.0)	
Relapse	12 (42.9)	4 (40.0)	—
Relapses cite			0.331
Intramedullary	3 (25.0)	3 (75.0)	
Extramedullary	6 (50.0)	1 (25.0)	
Both	3 (25.0)	0 (0)	

*Note*: Data are presented as *n* (%) unless indicated otherwise.

Abbreviations: aGVHD, acute graft versus host disease; cGVHD, chronic graft versus host disease; CMV, cytomegalovirus; EBV, Epstein–Barr virus.

### 3.2 Hematopoietic reconstitution

All patients showed neutrophil engraftment. The median time (11 vs. 11 days, *p* = 0.832) of neutrophil engraftment did not differ between HID‐HSCT and MSD‐HSCT groups. Except for two patients who developed PGF in the HID‐HSCT cohort, all patients showed platelet engraftment. The rate (92.9% vs. 100.0%, *p* = 1.000) and the median time (11 vs. 10 days, *p* = 0.166) of platelet engraftment did not differ significantly between the two groups. Except for two patients who died from TRM, all patients showed full‐donor chimerism 30 days after HSCT.

### 3.3 Infection and regimen‐related toxicities

The incidence of cytomegalovirus (CMV) reactivation was higher in the HID‐HSCT group than in the MSD‐HSCT group but without statistical significance (32.1% vs. 20.0%, *p* = 0.690). The incidence of Epstein–Barr virus reactivation was higher in the MSD‐HSCT group than in the HID‐HSCT group but without statistical significance (17.9% vs. 20.0%, *p* = 1.000). Ten patients in the HID‐HSCT group and two patients in the MSD‐HSCT group had pneumonia within 3 months after HSCT (35.7% vs. 20%, *p* = 0.453). Twelve and four patients in the HID‐HSCT and MSD‐HSCT groups had mild oropharyngeal mucositis, respectively (42.9% vs. 40%, *p* = 1.000). Moderate hemorrhagic cystitis only occurred in the HID‐HSCT group (10.7% vs. 0%, *p* = 0.552). No case of severe toxicity caused by regimens was found.

### 3.4 Graft‐versus‐host disease

**FIGURE 1:**
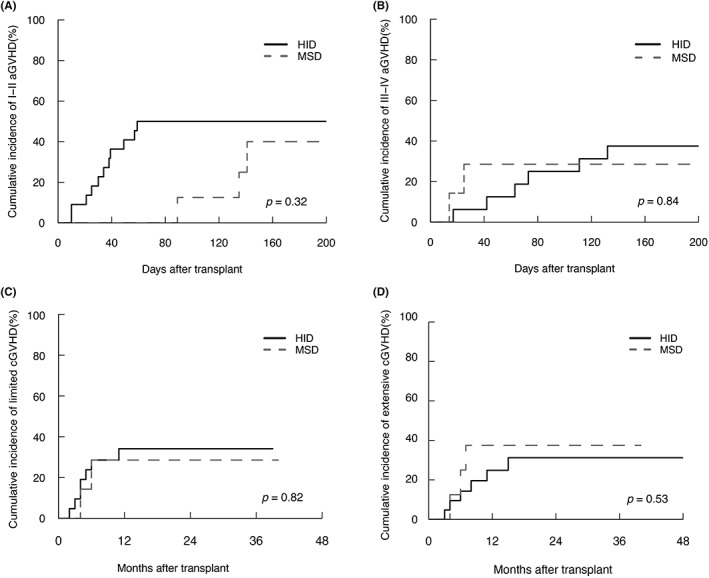
Cumulative incidence of acute graft‐versus‐host disease (aGVHD) in patients underwent allogeneic HSCT. The 200‐day cumulative incidences of grades I–II (aGVHD) (A) and grades III–IV aGVHD (B). The 2‐year cumulative incidences of limited chronic GVHD (cGVHD) (C) and extensive cGVHD (D) in haploidentical donor hematopoietic stem cell transplantation (HID‐HSCT) and matched sibling donor HSCT (MSD‐HSCT) groups.

Twelve patients in HID‐HSCT group and three patients in MSD‐HSCT group developed grades I–II aGVHD. The 200‐day cumulative incidences of grades I–II aGVHD was 50% (95% confidence interval [CI]: 47.59%–52.41%) in HID‐HSCT group and 40% (95% CI: 32.19%–47.81%, *p* = 0.32) in MSD‐HSCT group (Figure 1A). Six patients in HID‐HSCT group and two patients in MSD‐HSCT group developed grades III–IV aGVHD. The 200‐day cumulative incidences of grades III–IV aGVHD was 37.50% (95% CI: 34.33%–40.67%) in HID‐HSCT group and 28.57% (95% CI: 21.79%–35.35%, *p* = 0.84) in MSD‐HSCT group (Figure 1B). A total of 14 patients in the HID group developed cGVHD, with limited and extensive cGVHD in equal numbers. Two patients developed limited cGVHD and three patients developed extensive cGVHD in MSD‐HSCT. The 2‐year cumulative incidences of limited (34.13%, 95% CI: 31.82%–36.44% vs. 28.57%, 95% CI: 21.24%–35.91%, *p* = 0.82) or extensive (31.22%, 95% CI: 28.78%–33.65% vs. 37.50%, 95% CI: 30.28%–44.72%, *p* = 0.53) cGVHD did not differ between HID‐HSCT and MSD‐HSCT groups (Figure 1C,D).

### 3.5 Survival and relapse

**FIGURE 2:**
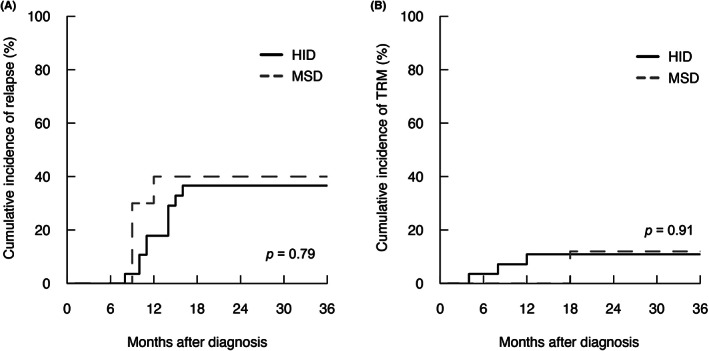
Cumulative incidence of relapse and transplantation‐related mortality in patients underwent alloHSCT. (A) The 2‐year cumulative incidence of relapse were 36.61% (95% CI: 34.85%–38.37%) and 40.0% (95% CI: 34.68%–45.32%) in haploidentical donor hematopoietic stem cell transplantation (HID‐HSCT) and matched sibling donor HSCT (MSD‐HSCT) group respectively (*p* = 0.79). (B) The 2‐year cumulative incidence of transplantation‐relative mortality was not significantly different between HID‐HSCT and MSD‐HSCT group (10.89%, 95% CI: 10.17%–11.61% vs. 12%, 95% CI: 8.96%–15.04%, *p* = 0.91).

**FIGURE 3:**
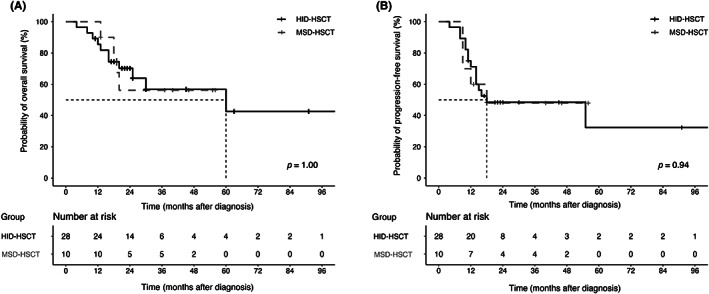
Kaplan–Meier curve for overall survival (OS) and progression‐free survival (PFS) in patients of allogeneic‐HSCT. (A) The estimated 2‐year OS rates was 70.3% (95% CI: 54.9%–90.0%) in haploidentical donor hematopoietic stem cell transplantation (HID‐HSCT) cohort and 56.2% (95% CI: 31.6%–100%) in matched sibling donor HSCT (MSD‐HSCT) cohort (*p* = 1.00). (B) The estimated 2‐year PFS rates were 48.5% (95% CI: 32.8%–71.6%) in HID‐HSCT group and 48.0% (95% CI: 24.6%–93.8%) in MSD‐HSCT group (*p* = 0.94), respectively.

Twelve patients in the HID‐HSCT group and four patients in the MSD‐HSCT group experienced a relapse of malignancy. The 2‐year CIR (36.61%, 95% CI: 34.85%–38.37% vs. 40.0%, 95% CI: 34.68%–45.32%) did not vary significantly between the two groups (*p* = 0.79, Figure 2A). A total of two patients in HID‐HSCT group and one patient in MSD‐HSCT group died of transplant‐related complications, with one patient in each group dying from grades III to IV aGVHD. The 2‐year cumulative incidences of TRM were 10.89% (95% CI: 10.17%–11.61%) in HID‐HSCT group and 12% (95% CI: 8.96%–15.04%) in MSD‐HSCT group (*p* = 0.91, Figure 2B), respectively. Since MSD‐HSCT group had a smaller number of patients, the TRM incidence was slightly higher than that of HID‐HSCT group. In a randomized multicenter study comparing MSD‐HSCT with HID‐HSCT in adults with Ph‐ALL in CR1, the 3‐year cumulative TRM were 13% and 11% (*p* = 0.84) in respective group.^17^ According to a retrospective study of allo‐HSCT outcomes in adults with standard‐risk ALL in CR1, the 5‐year cumulative TRM were 16.4% and 11.6% for HID‐HSCT and MSD‐HSCT, respectively.^18^ Our results were comparable with parallel studies. Eleven (39.29%) patients in the HID‐HSCT group and four (40%) patients in the MSD‐HSCT group died, with causes including infection (four cases in HID‐HSCT and two cases in MSD‐HSCT), respiratory/circulatory failure (four cases in HID‐HSCT and one case in MSD‐HSCT), and relapse (three cases in HID‐HSCT and one case in MSD‐HSCT). The estimated 2‐year OS rates was 70.3% (95% CI: 54.9%–90.0%) in the HID‐HSCT group and 56.2% (95% CI: 31.6%–100%) in the MSD‐HSCT group (*p* = 1.00, Figure 3A). The estimated 2‐year PFS rates were 48.5% (95% CI: 32.8%–71.6%) and 48.0% (95% CI: 24.6%–93.8%) in the HID‐HSCT and MSD‐HSCT groups, respectively, showing no significant difference (*p* = 0.94, Figure 3B), with a median PFS of 18 months after diagnosis.

### 3.6 Univariate and multivariate analyses

In order to identify potential influencing factors, basic information and clinical outcome information in Tables 1 and 2 were included in the Cox analysis. Continuous variables were converted into categorical variables by using the median as the dividing line such as age, PLT engraftment time and neutrophil engraftment time. The univariate analysis showed that B symptoms, Eastern Cooperative Oncology Group score 2, International Prognostic Index score 3–4, mediastinal mass at diagnosis, PGF, cGVHD, and relapse affected OS in patients with T‐LBL who underwent allo‐HSCT. Nevertheless, these variables did not differ significantly in the multivariate analysis. Risk factors significantly affecting PFS included B symptoms, cGVHD, PGF, platelet engraftment time >11 days, and a PET/CT‐positive status before HSCT in the univariate analysis. However, only the PET/CT‐positive status before HSCT (*p* = 0.0367, HR = 2.99) was an independent risk factor for PFS in the multivariate analysis (Table 4).

## 4 DISCUSSION

The prognosis of T‐LBL is poor. Although no standard therapy for T‐LBL exists, chemotherapy combined with transplantation provides survival advantages and, occasionally, improves the prognosis.^12^, ^33^, ^34^ Among transplantation approaches, allo‐HSCT lowers the risk of relapse significantly greater than auto‐HSCT, improving PFS and survival.^11^, ^35^ HID‐HSCT is occasionally used to treat T‐LBL/ALL, and studies on selection criteria and comparison of HID‐HSCT and MSD‐HSCT are limited. This retrospective study offered novel evidence of their effectiveness and safety being comparable.

Patients with T‐LBL treated with allo‐HSCT have a wide range of survival results, which may be attributed to differences in patient characteristics. In a multicenter study, PFS of 75% at 3 years, not significantly differing from auto‐HSCT.^12^ Another multicenter study enrolled 21 patients with T‐LBL who received HSCT (16 of them underwent allo‐HSCT), the 5‐year OS were 56.3% in the whole cohort.^36^ A retrospective analysis showed an OS of 70% and a PFS of 65% at 3 years in 25 patients with T‐LBL who underwent T‐cell‐replete HID‐HSCT.^20^ An American study involving 28 patients who underwent HSCT, including auto‐HSCT and allo‐HSCT, showed an OS of 49% and a PFS of 46% at 3 years.^37^ In a single‐center study, the 3‐year OS and PFS were both 56.2% of 16 patients undergoing allo‐HSCT.^38^ In this study, the survival outcome showed similar estimated 2‐year OS (70.3% vs. 56.2%, *p* = 1.000) and PFS (48.5% vs. 48%, *p* = 0.94) and the same median PFS time (18 months). Our results were consistent with those of previous studies.

These two procedures are also similar in safety. In this study, hemorrhagic cystitis occurred only in the HID‐HSCT group, because of the conditioning regimens, but all of the cases were mild. CMV and Epstein–Barr virus reactivation rates did not differ between the two groups; however, the HID‐HSCT group had a slightly greater CMV activation rate compared to the MSD‐HSCT group. Atibordee et al. summarized several risk factors for CMV infection in transplantation recipients, such as ATG, ALG, high‐dose MMF, and other immunosuppressive drugs.^39^ Compared to the conditioning regimen and prevention of GVHD in patients with MSD‐HSCT, immunosuppressive strategies for patients with HID‐HSCT are stronger. In clinical practice, care should be taken to monitor the virus reactivation status of patients, and antiviral medication should be administered promptly if virus reactivation occurs.

The results of hematopoietic reconstitution were statistically equivalent in the two groups. Two patients in the HID‐HSCT group experienced PGF, one of whom died from severe infection with respiratory failure 33 days after HSCT. This patient had received <5 × 10^6^/kg of CD34^+^ infusion. Risk factors for primary PGF include a low dosage of CD34^+^ cell infusion, donor‐specific antibodies, GVHD, CMV infection, iron overload, and splenomegaly.^40^, ^41^ Patients with PGF have worse OS and PFS.^28^, ^41^, ^42^, ^43^ In some clinical trials of ALL, HID‐HSCT showed marginally higher TRM or non‐relapse mortality but comparable and even slightly superior PFS or disease‐free survival compared to MSD‐HSCT.^17^, ^18^ In our multivariate analysis, PGF was not independent risk factor. Although a limited number of patients developing PGF or TRM did not affect the overall statistical outcome of patients, these events should be paid attention to during transplantation to reduce the risk of complications.

**FIGURE 4:**
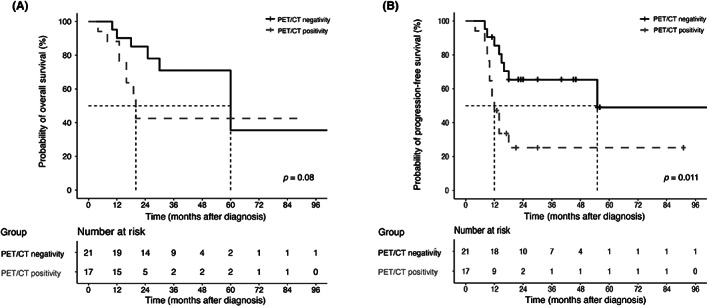
Kaplan–Meier curve for overall survival (OS) and progression‐free survival (PFS) in patients grouped by positron emission tomography/computed tomography (PET/CT) evaluation. (A) The estimated 2‐years OS rates was 85.2% (95% CI: 71.07%–100%) in PET/CT negative cohort and 42.5% (95% CI: 23.6%–76.6%) in PET/CT positive cohort (*p* = 0.08), respectively. (B) The estimated 2‐years PFS rates was 65.3% (95% CI: 47.5%–89.8%) in PET/CT negative group and 25.2% (95% CI: 10.3%–61.5%) in PET/CT positive group (*p* = 0.011).

We identified multiple univariate variables associated with the prognosis in the Cox analysis of OS and PFS (Tables 3 and 4). A PET/CT‐positive status before HSCT was an independent hazard factor for PFS (multivariate *p*‐value = 0.0367; hazard ratio = 2.99). This finding suggests that patients with T‐LBL who attain a PET/CT‐negative status before transplantation could experience better outcomes (Figure 4).

**TABLE 3:** Univariate and multivariate analysis of overall survival in allo‐HSCT.

Characteristics	Univariate *p* value	HR (95% CI)	Multivariate *p* value	HR (95% CI)
B symptom	0.004	5.08 (1.66–15.54)	0.064	5.09 (0.91–28.45)
ECOG = 2	0.007	4.94 (1.55–15.73)	0.4016	0.43 (0.06–3.14)
IPI = 3–4	0.02	4.14 (1.26–13.64)	0.5791	0.6 (0.1–3.58)
Mediastinal mass	0.032	9.55 (1.21–75.48)	0.0754	9.1 (0.8–103.75)
PGF occurrence	0.004	10.59 (2.11–53.15)	0.4054	2.15 (0.36–12.97)
cGVHD occurrence	0.033	0.31 (0.1–0.91)	0.0828	0.2 (0.03–1.24)
Relapse	0.026	3.68 (1.17–11.59)	0.1164	3.18 (0.75–13.44)
MSD versus HID	0.992	1.01 (0.31–3.22)	—	—

Abbreviations: 95% CI, 95% confidence interval; cGVHD, chronic graft versus host disease; ECOG, ECOG score; HID, haploidentical donor HSCT; HR, hazard ratio; IPI, the International Prognostic Index; MSD, matched sibling donor HSCT; PGF, poor graft function.

**TABLE 4:** Univariate and multivariate analysis of progression‐free survival in allo‐HSCT.

Characteristics	Univariate *p* value	HR (95% CI)	Multivariate *p* value	HR (95% CI)
B symptom	0.039	2.64 (1.05–6.61)	0.3789	1.65 (0.54–5.03)
cGVHD occurrence	0.044	0.38 (0.15–0.97)	0.2713	0.56 (0.2–1.58)
PGF occurrence	0.045	4.6 (1.03–20.5)	0.9715	0.97 (0.18–5.13)
PLT engraftment time >11 days	0.034	2.76 (1.08–7.04)	0.1339	2.36 (0.77–7.24)
PET/CT positive	0.015	3.11 (1.25–7.74)	0.0367	2.99 (1.07–8.33)
MSD versus HID	0.952	0.97 (0.35–2.68)	—	—

Abbreviations: 95% CI, 95% confidence interval; cGVHD, chronic graft versus host disease; HID, haploidentical donor HSCT; HR, hazard ratio; MSD, matched sibling donor HSCT; PET/CT positive, Deauville score >3; PGF, poor graft function; PLT, platelet.

However, the role of PET/CT in T‐LBL is debatable. Dai et al. discovered that PET/CT after allo‐HSCT was a powerful predictor of PFS but not of OS in 67 patients with LBL.^44^ Sun et al. demonstrated that PET/CT before HSCT could not predict PFS or OS but that the post‐HSCT PET/CT‐positive group had a greater risk compared to the PET/CT‐negative group among 21 consecutive patients with relapsed or refractory T‐LBL who underwent HSCT.^45^ This paradoxical outcome may be attributable to differing time points of PET/CT evaluation before HSCT. We performed PET/CT assessment after the completion of chemotherapy, while Sun et al. performed PET/CT assessments 2–10 weeks before transplantation, thereby possibly including patients who were still on chemotherapy without achieving remission. Due to a paucity of relevant research, the importance of PET/CT before and after transplantation in patients with T‐LBL remains underexplored, and prospective studies are required for further investigation.

In summary, we demonstrated the effectiveness of allo‐HSCT in treating T‐LBL. HID‐HSCT is a viable alternative that can yield equivalent survival outcomes compared to MSD‐HSCT. Achievement of a PET/CT‐negative status before allo‐HSCT may contribute to better survival of patients with T‐LBL. A bigger, prospective study is required to further validate our findings.

## AUTHOR CONTRIBUTIONS

**Ruowen Wei:** Data curation (equal); formal analysis (equal); investigation (equal); methodology (equal); software (equal); validation (equal); visualization (equal); writing—original draft (equal). **Jun Fang:** Conceptualization (lead); data curation (lead); formal analysis (lead); investigation (lead); methodology (equal); project administration (lead); supervision (equal); writing—review and editing (lead). **Wei Shi:** Conceptualization (equal); methodology (equal); project administration (equal); writing—review and editing (equal). **Xuan Lu:** Data curation (equal); resources (equal). **Yingying Wu:** Formal analysis (equal); methodology (equal); resources (equal); visualization (equal). **Shan Jiang:** Data curation (equal); resources (equal). **Ao Zhang:** Data curation (equal). **Shanshan Liao:** Data curation (equal); resources (equal); software (equal). **Chunxia Qin:** Resources (equal); software (equal). **Guohui Cui:** Conceptualization (lead); formal analysis (lead); methodology (lead); project administration (lead); supervision (equal); writing—review and editing (lead). **Linghui Xia:** Conceptualization (lead); formal analysis (lead); project administration (lead); supervision (lead); writing—review and editing (lead).

## FUNDING INFORMATION

None.

## CONFLICT OF INTEREST STATEMENT

There are no conflicts of interest to report.

## Data Availability

All data, models, or code generated or used during the study are available from the corresponding author by request.
